# Impact of Percutaneous Endoscopic Decompression Versus Open Laminectomy on Postoperative Acute Urinary Retention: A Large-Scale Real-World Data Analysis

**DOI:** 10.3390/jcm15124519

**Published:** 2026-06-11

**Authors:** Sz-En Lee, Jian-Ri Li, Cheng-Ying Lee, Hsi-Kai Tsou, Cheng-Ta Chou, Ting-Hsien Kao

**Affiliations:** 1 Department of Urology, Taichung Veterans General Hospital, Taichung City 40705, Taiwan; jordan9923@gmail.com (S.-E.L.); fisherfishli@yahoo.com.tw (J.-R.L.); 2 Department of Neurosurgery, Neurological Institute, Taichung Veterans General Hospital, Taichung City 40705, Taiwan; twendospine@gmail.com (C.-Y.L.); tsouhsikai@gamil.com (H.-K.T.); 3 Department of Post-Baccalaureate Medicine, College of Medicine, National Chung Hsing University, Taichung City 40227, Taiwan; 4 Department of Neurology, Neurological Institute, Taichung Veterans General Hospital, Taichung City 40705, Taiwan; ctchou57@gmail.com; 5 Division of Minimally Invasive Neurosurgery, Neurological Institute, Taichung Veterans General Hospital, Taichung City 40705, Taiwan

**Keywords:** percutaneous endoscopic lumbar surgery, laminectomy, acute urinary retention, benign prostatic hyperplasia

## Abstract

**Background/Objectives**: To compare the incidence of postoperative acute urinary retention (AUR) between traditional open laminectomy and percutaneous endoscopic lumbar surgery (PELS) using a large-scale real-world database, with specific stratification by urologic status, age, and sex. **Methods**: A retrospective, propensity score-matched analysis was conducted using the TriNetX Global Health Research Network (2015–2024). Adult patients undergoing PELS were compared to those undergoing open laminectomy. To rule out the confounding effect of routine intraoperative catheterization, the primary outcome was defined as de novo AUR occurring between 24 h and 3 months postoperatively. Subgroup analyses were performed for patients with benign prostatic hyperplasia (BPH), females, and age-stratified cohorts (<70 vs. ≥70 years). This study was approved by the Institutional Review Board (IRB/REC: CE25727C) and conducted under a waiver of informed consent. **Results**: In the matched cohorts of non-BPH males, females, and patients aged < 70 years, PELS was associated with a statistically significant reduction in AUR risk (Hazard Ratios: 0.445, 0.649, and 0.403, respectively) compared to open surgery. However, in males with BPH, the protective benefit of the endoscopic technique was attenuated and did not reach statistical significance (*p* = 0.0744), suggesting the study was underpowered for this subgroup or that baseline obstruction remains a dominant risk factor. **Conclusions**: Percutaneous endoscopic lumbar surgery was associated with a significantly lower risk of postoperative AUR compared to open laminectomy, particularly in patients without preexisting urologic obstruction. This benefit is likely attributable to minimized tissue trauma and the anti-inflammatory effects of continuous saline irrigation. However, in patients with BPH, baseline pathology outweighs surgical factors, necessitating medical prophylaxis regardless of the surgical approach.

## 1. Introduction

Acute urinary retention (AUR) is a frequent early postoperative complication following lumbar spine surgery, with reported incidence rates ranging from 6% to over 20% depending on the surgical approach. This complication can significantly impede recovery by delaying postoperative mobilization, prolonging hospitalization, and increasing the risk of catheter-associated urinary tract infections. However, the risk of retention is not uniformly distributed across all patients. Large-scale meta-analyses have firmly established that patient-specific factors—specifically advanced age and male sex—are critical independent predictors of this adverse event [1].

The pathophysiology of postoperative retention in this population is intimately linked to the anatomical proximity of the neural elements. Although bladder storage and voiding are primarily regulated by the sacral micturition centers, the descending autonomic pathways that connect supraspinal control to these centers must traverse the lumbar spinal canal. As described in fundamental models of spinal cord compression, mechanical deformation or surgical manipulation at this level can disrupt these delicate autonomic signals, precipitating sphincter dysfunction and retention [2].

Surgical invasiveness is hypothesized to play a pivotal role in this mechanism. Traditional posterior lumbar surgeries—such as open laminectomy—have long been the standard approach due to their broad anatomical exposure, excellent direct visualization of neural elements, and widespread familiarity among surgeons. However, these traditional techniques require extensive muscle stripping and prolonged retraction of paraspinal tissues. This degree of invasiveness has been associated with significant drawbacks, including increased neural irritation, longer operative times, and greater soft-tissue damage, all of which can exacerbate postoperative autonomic dysfunction. Conversely, percutaneous endoscopic lumbar surgery (PELS) aims to mitigate these insults. High-quality evidence from pragmatic trials has demonstrated that PELS approaches, such as microdecompression, achieve equivalent clinical efficacy to open surgery while significantly reducing soft-tissue damage and hospital stays [3].

Prospective studies have demonstrated that full-endoscopic procedures achieve clinical outcomes comparable to those of microsurgery while offering superior benefits in minimizing tissue trauma and accelerating functional recovery [4].

Despite these established benefits of PELS regarding tissue preservation, comparative data specifically addressing AUR rates remain inconsistent. This inconsistency is likely driven by the heterogeneity of patient baselines. For instance, benign prostatic hyperplasia (BPH) creates a physiological outlet obstruction in males that may override the benefits of a less invasive surgical technique. Similarly, the aging bladder exhibits different contractility profiles compared to younger cohorts, and gender-specific pelvic anatomy further complicates the risk landscape.

The objective of this study is to compare the incidence of postoperative acute urinary retention between traditional open lumbar surgery and percutaneous endoscopic lumbar surgery. To ensure clinical precision, we performed dedicated subgroup analyses stratified by urologic status (BPH vs. non-BPH), age (<70 vs. ≥70 years), and sex, allowing for a clearer understanding of whether PELS confers differential protective effects across these distinct populations.

## 2. Materials and Methods

### 2.1. Data Source and Study Design

The data used in this study were collected on 7 January 2026 from the TriNetX Global Health Research Network, which provides access to electronic medical records (diagnoses, procedures, medications, laboratory values, genomic information) from approximately 91,118,422 patients across 172 healthcare organizations. This federated research database provides longitudinal patient-level data, allowing for the retrospective analysis of large-scale clinical cohorts. The architectural framework, validity, and utility of this federated real-world database for tracking longitudinal patient cohorts across diverse health systems have been extensively detailed previously [5]. Furthermore, the specific application of the TriNetX platform to evaluate spine surgery outcomes and capture postoperative complications via propensity score matching has been well-established in recent surgical literature [6]. The reporting of this observational study conforms to the Strengthening the Reporting of Observational Studies in Epidemiology (STROBE) guidelines (see Supplementary STROBE Checklist).

### 2.2. Study Population

The selection process followed a structured, sequential attrition pipeline to isolate patients with homogeneous baseline characteristics and ensure high data completeness. The index date was defined as the day of the qualifying lumbar spine surgery. The clinical cohort was established through the following step-by-step filtering process:

- Initial Database Screening: Adult patients (aged >18 years) who underwent lumbar spine decompression procedures between 1 January 2015 and 31 December 2024 were initially pulled from the global network. Because this study utilized a pre-existing federated database, the sample size was determined by the total available patient cohort that met our strict eligibility criteria within the network, rather than an a priori power calculation.
- Exclusion of Pre-existing Urologic Conditions: To prevent the confounding influence of chronic lower urinary tract symptoms (LUTS) or preexisting bladder failure on our primary outcome, we systematically excluded any patient with a documented historical diagnosis of neurogenic bladder, baseline preoperative urinary retention, or chronic indwelling catheterization documented prior to or on the index date.
- Exclusion of Malignancies (Neoplasms): To eliminate mechanical or functional bladder outlet obstruction secondary to oncological processes or pelvic radiation, all patients with a pre-existing history of any neoplasm were strictly excluded from the study population.
- Requirement for Follow-up Completeness: Patients who lacked at least 30 days of continuous post-operative health record data within the federated network were excluded to minimize attrition bias and preserve longitudinal data integrity.

Following the application of these strict eligibility criteria, the remaining unselected patients were stratified into the four target clinical phenotypes: Non-BPH Males, Males with BPH, Females, and Age-stratified groups (<70 vs. ≥70 years). Within each subpopulation, 1:1 nearest-neighbor propensity score matching (PSM) was executed.

### 2.3. Exposure Definitions and Stratification

Patients were categorized into two mutually exclusive surgical exposure cohorts based on a validated, granular billing and procedural coding strategy. To prevent cohort cross-contamination and guarantee the structural validity of our comparisons, a hybrid algorithm combining the AMA Current Procedural Terminology (CPT) and the ICD-10 Procedure Coding System (ICD-10-PCS) was utilized:

- Traditional Open Surgery Cohort: Patients were identified via standard 5-digit AMA CPT codes for open lumbar decompression aggregated under the TriNetX hierarchical master parent node (Concept ID: 1009381; Posterior Extradural Laminotomy or Laminectomy for Exploration/Decompression of Neural Elements or Excision of Herniated Intervertebral Disks Procedures). To ensure an exhaustive capture of open posterior decompression, this master node encompassed all primary, multi-level, and re-exploration standard codes, specifically: CPT 63001, 63003, 63005, 63011, 63012, 63015, 63016, 63017, 63020, 63030, 63035, 63040, 63042, 63043, 63044, 63045, 63046, 63047, 63048, 63050, 63051, 63052, and 63053.
- Percutaneous Endoscopic Lumbar Surgery (PELS) Cohort: Patients were identified exclusively using the multiaxial ICD-10-PCS code 00NY4ZZ (Release Lumbar Spinal Cord, Percutaneous Endoscopic Approach).

Because standard CPT billing codes for spinal decompression (e.g., CPT 63030 or 63047) frequently conflate traditional open surgeries, micro-tubular retractor systems, and full percutaneous endoscopic techniques under the same code, a standard CPT query alone is inadequate to isolate pure endoscopic cohorts.

To resolve this limitation, our validation algorithm isolated the PELS cohort using the 5th character identifier (“4”) within the ICD-10-PCS framework, which explicitly mandates a true percutaneous endoscopic approach. Patients capturing standard decompression CPT codes who concurrently carried this endoscopic ICD-10-PCS code were allocated to the PELS cohort. Conversely, those lacking the endoscopic modifier were finalized into the Traditional Open cohort, ensuring absolute separation of the surgical techniques. However, because administrative billing codes are subject to human error, some misclassification risk remains if coders conflated tubular microscopic retractors with true percutaneous endoscopy.

To evaluate the differential impact of surgical technique across vulnerable populations, the cohort was stratified into three distinct categories:

- Urologic Status: Patients were divided into a BPH group and a non-BPH group (males only) based on preoperative diagnosis codes.
- Age: Patients were stratified into those aged < 70 years and those aged ≥ 70 years.
- Sex: A dedicated female cohort was analyzed separately to isolate gender-specific anatomical risks.

### 2.4. Primary Outcome

The primary outcome was the incidence of acute urinary retention (AUR). AUR was identified by the presence of the diagnostic code for retention (ICD-10: R33) and/or procedural codes indicating Foley catheter insertion (CPT 51702, 51703). Crucially, to eliminate selection bias and the confounding risk of misclassifying routine, intraoperative prophylactic catheterizations as adverse events, the observation window was strictly set from 24 h to 3 months following the index surgery. Any retention codes or catheterizations documented on the day of surgery (Day 0 to <24 h) were excluded from the primary outcome analysis, ensuring that only true, symptomatic postoperative retention events were captured. This temporal window systematically filters out routine prophylactic intraoperative Foley placements and standard Post-Anesthesia Care Unit (PACU) catheterizations, ensuring that we are capturing de novo, symptomatic retention events that occurred after the patient was expected to void independently.

### 2.5. Statistical Analysis

All statistical analyses were executed within the advanced TriNetX Analytics platform environment. To control for confounding and selection bias inherent to retrospective observational studies, propensity score matching (PSM) was implemented to balance baseline covariates between the traditional open and PELS cohorts. Matching performance was evaluated using a 1:1 nearest-neighbor matching algorithm with a strict caliper width of 0.1 pooled standard deviations. This propensity score matching (PSM) technique successfully balanced the groups across all measured baseline covariates. The utilization of these specific propensity score methods and strict caliper constraints serves as a robust methodological standard to drastically reduce selection bias and minimize the effects of overt confounding in retrospective observational database cohorts [7]. This approach ensures that baseline balance across a high-dimensional set of covariates is mathematically verified before calculating treatment effects [8]. Covariate balance before and after matching was systematically verified using Standardized Mean Differences (SMD), where an SMD < 0.1 indicated negligible residual bias.

Prior to comparative analysis, continuous variables—specifically age—were evaluated for distributional normality using the Kolmogorov–Smirnov test alongside visual inspection of quantile-quantile (Q-Q) plots. Given the large-scale nature of our pre-matched dataset, standard normality significance tests are highly sensitive to trivial deviations; therefore, primary reliance was placed on standard deviation limits, skewness, and visual plotting. For normally distributed continuous data, independent two-sample *t*-tests were performed, while the Mann–Whitney *U* test was designated for non-normally distributed metrics. Categorical clinical characteristics were compared using Pearson’s chi-squared test or Fisher’s exact test where appropriate.

Time-to-event outcomes for acute urinary retention (AUR) were mapped using Kaplan–Meier survival curves, and differences between surgical approaches were evaluated via the log-rank test. The underlying mathematical assumptions of the Cox proportional hazards models were explicitly evaluated by testing the time-dependency of the treatment effects (proportional hazards assumption tests). For subgroups where the proportionality assumption held true (e.g., non-BPH males, patients aged < 70, and females), a single, constant Hazard Ratio (HR) with 95% confidence intervals (CI) was calculated. For the cohorts where the proportional hazards assumption was statistically violated (e.g., patients aged ≥ 70, *p* = 0.0270), the non-constant hazard behavior was further analyzed using log-log survival curves to identify where the hazard functions diverged or converged over the 3-month observation window, ensuring that the attenuated global HR was interpreted safely as a weighted average clinical effect. Two-sided *p* < 0.05 was considered statistically significant for all tests.

To quantify the potential impact of unmeasured confounding, we calculated the E-value for our primary cohort. The E-value estimates the minimum strength of association that an unmeasured confounder would need to have with both the treatment and the outcome to fully explain away a specific treatment-outcome association, conditional on the measured covariates.

Finally, to address instances where the proportional hazards assumption was statis-tically violated (Schoenfeld residual test *p* < 0.05), a Restricted Mean Survival Time (RMST) analysis was conducted. Evaluated at a pre-specified horizon of 90 days, the RMST pro-vides a model-free measure of the mean event-free time accrued over the follow-up window (see Supplementary Methods and Supplementary Table S1). Furthermore, to control the family-wise error rate across our five pre-specified clinical subgroups, a Bonferroni correc-tion was applied to the log-rank *p*-values, establishing an adjusted significance threshold of α = 0.01 (see Supplementary Table S2 and Supplementary Interpretation).

## 3. Results

### 3.1. Patient Characteristics and Propensity Score Matching

After applying exclusion criteria, the study population was stratified into four distinct analytic cohorts: Males without Benign Prostatic Hyperplasia (BPH), Males with BPH, Females, and Age-stratified groups (<70 and ≥70 years). Baseline characteristics for these groups prior to and following propensity score matching (PSM) are detailed in Table 1, Table 2, Table 3, Table 4 and Table 5. Before matching, significant heterogeneity existed between the intervention groups. For instance, in the Age ≥ 70 cohort, patients undergoing Traditional Open Surgery had a significantly higher prevalence of cerebrovascular disease compared to the Percutaneous Endoscopic Lumbar Surgery (PELS) group (*p* < 0.001). Similarly, in the Non-BPH Male cohort, racial distribution and metabolic comorbidities differed markedly between approaches. Following 1:1 propensity score matching, excellent covariate balance was achieved across all subgroups. The Standardized Mean Differences (SMD) for all matched variables—including age, race, BMI, and major comorbidities (hypertension, diabetes, ischemic heart disease)—were consistently below 0.1, indicating negligible residual bias.

- Non-BPH Male Cohort: The matched analysis included approximately 2398 patients per group, with a mean age of 61.0 years.
- BPH Male Cohort: This high-risk subgroup (approx. N = 232 per group) was notably older, with a mean age of 71.6 years, and exhibited a higher burden of age-related comorbidities.
- Female Cohort: The matched female population (approx. N = 2144 per group) had a mean age of 64.7 years.
- Age-Stratified Cohorts: The younger cohort (<70 years) had a mean age of 52.9 years, while the elderly cohort (≥70 years) had a mean age of 73.3 years.

### 3.2. Incidence of Acute Urinary Retention and Subgroup Analysis

The overall incidence of postoperative acute urinary retention (AUR) varied significantly by patient phenotype and surgical approach.

#### 3.2.1. Urologic Stratification

**Figure 1:**
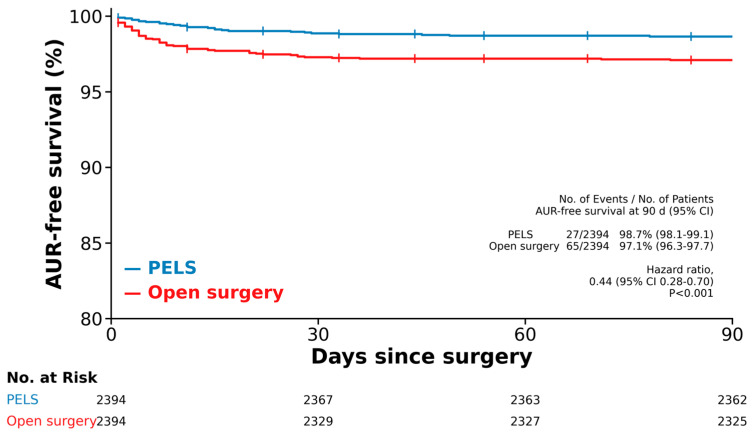
Survival curve of the non-BPH male cohort.

**Figure 2:**
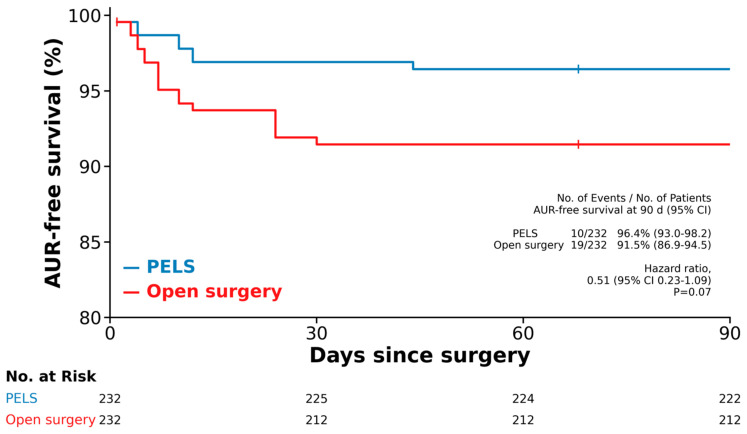
Survival curve of the BPH male cohort.

- Non-BPH Males: In the comparative analysis of 4788 patients, the PELS cohort demonstrated a significantly lower incidence of AUR compared to the Traditional open surgery cohort, with absolute risks of 1.128% and 2.715%, respectively (Risk Difference: −1.587%; *p* < 0.0001). Patients in the PELS group experienced an approximately 59% reduction in the odds of AUR (OR: 0.409; 95% CI: 0.26–0.643) and a 55.5% lower instantaneous hazard (HR: 0.445; 95% CI: 0.284–0.697) compared to the Traditional open surgery cohort. Sensitivity analysis to quantify the potential for unmeasured confounding in this primary cohort yielded an E-value of 3.92 for the point estimate and 2.22 for the upper confidence limit. This suggests that an unmeasured confounder would need to be associated with both the selection of PELS and the incidence of AUR by a risk ratio of 3.92 each to fully negate the observed risk reduction, indicating that this association is robust against moderate hidden bias. Kaplan–Meier survival analysis confirmed superior event-free outcomes for the PELS approach (Log-Rank *p* = 0.0003), with end-of-window survival probabilities of 98.662% for PELS and 97.101% for the Traditional open surgery cohort, though a significant proportionality test (*p* = 0.0317) suggests the relative risk between these procedures may fluctuate over the post-operative period (Figure 1).

- BPH Patients: The PELS cohort (N = 232) showed a lower absolute risk of acute urinary retention (AUR) compared to the Traditional open surgery cohort (N = 232), with rates of 4.31% and 8.19%, respectively. Although the PELS cohort was associated with a nearly 50% reduction in the odds of AUR (OR: 0.505; 95% CI: 0.23–1.111) and a lower instantaneous hazard (HR: 0.505; 95% CI: 0.235–1.086), these findings did not reach statistical significance (*p* = 0.0843 for risk difference; *p* = 0.0744 for log-rank test). Kaplan–Meier analysis estimated event-free survival probabilities at the end of the time window to be 95.502% for the PELS group and 91.468% for the Traditional open surgery group. The proportionality test for this subgroup was non-significant (*p* = 0.2493), indicating that the hazard rates remained relatively constant over time, though the overall smaller sample size and privacy-related data rounding likely limited the statistical power to detect a definitive difference (Figure 2).

#### 3.2.2. Age-Stratified Analysis

**Figure 3:**
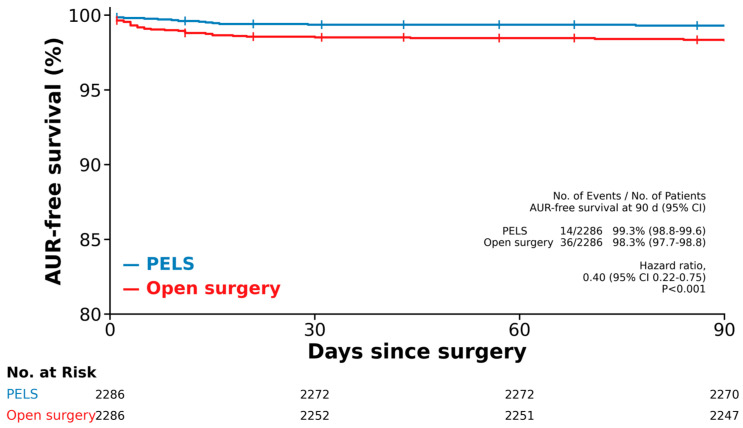
Survival curve of patients in the Age < 70 years cohort.

**Figure 4:**
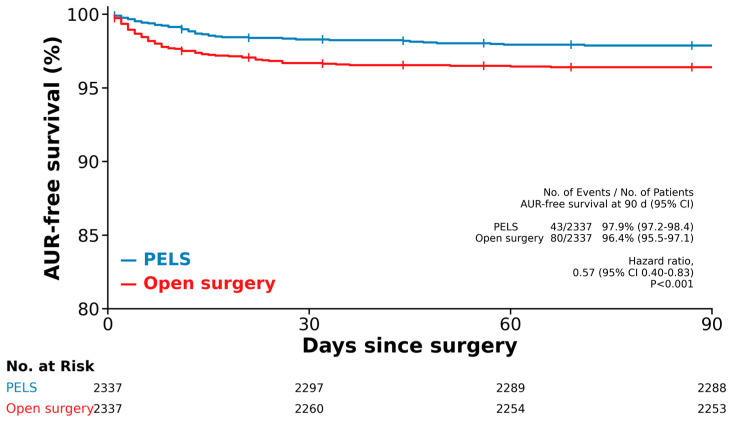
Survival curve of patients in the Age ≥ 70 years cohort.

- Age < 70 years: In the subgroup of patients under 70 years of age (N = 4572), the PELS group was associated with a statistically significantly lower AUR risk, with a rate of 0.612% compared to 1.575% in the Traditional open surgery cohort (Risk Difference: −0.962%; *p* = 0.0018). This younger cohort undergoing PELS experienced a 61.5% reduction in the odds of AUR (OR: 0.385; 95% CI: 0.207–0.716) and a significantly lower hazard (HR: 0.403; *p* = 0.0028). Notably, the proportionality test for the under-70 cohort was non-significant (*p* = 0.7299), indicating a stable treatment effect over time (Figure 3).

- Age ≥ 70 years: For the cohort of patients aged 70 years and older (N = 4674), the PELS group was associated with a significantly lower risk of AUR compared to the Traditional open surgery cohort. The absolute risk in the PELS group was 1.84% (43 events) versus 3.423% (80 events) in the Traditional open surgery group, resulting in a significant absolute risk reduction of 1.583% (*p* = 0.0007). Relative measures further confirmed this benefit, with an Odds Ratio of 0.529 (95% CI: 0.363–0.77) and a Hazard Ratio of 0.574 (95% CI: 0.396–0.832; *p* = 0.0030), indicating that older patients undergoing percutaneous procedures had a 42.6% lower hazard of AUR. While the Log-Rank test confirmed superior event-free survival for the PELS group (*p* = 0.0030), a significant proportionality test (*p* = 0.0270) suggests that the protective effect of the PELS approach compared to traditional open surgery may vary in magnitude over the post-operative course in this older demographic (Figure 4).

#### 3.2.3. Female Group

**Figure 5:**
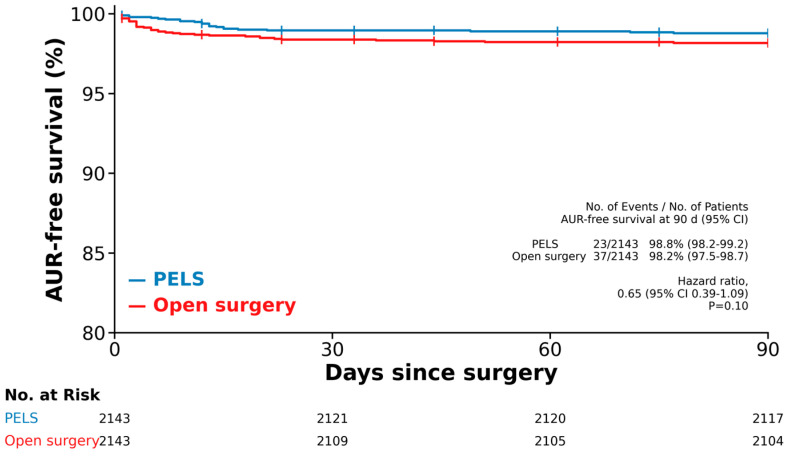
Survival curve of the Female patient cohort.

- In the analysis of female patients (N = 4286), the PELS approach demonstrated a trend toward lower AUR rates, though it did not reach statistical significance. The absolute risk was 1.073% (23 events) in the PELS cohort compared to 1.727% (37 events) in the traditional open surgery cohort (Risk Difference: −0.653%; *p* = 0.0687). The relative risk reduction followed a similar pattern, with an Odds Ratio of 0.618 (95% CI: 0.366–1.043) and a Hazard Ratio of 0.649 (95% CI: 0.386–1.092). Kaplan–Meier survival analysis yielded a Log-Rank *p*-value of 0.1005, indicating that the difference in time-to-event outcomes between the two surgical groups was not statistically significant for women. The proportionality test for this subgroup was also non-significant (*p* = 0.0630), suggesting a relatively constant, albeit statistically non-significant, risk profile throughout the observation window (Figure 5).

**Figure 6:**
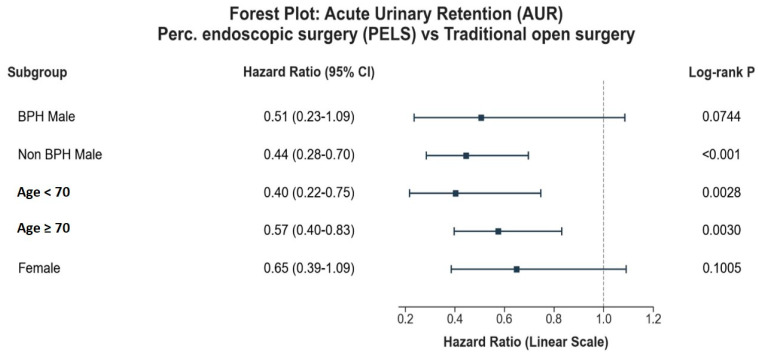
Forest plot of subgroup analysis.

To better compare the hazard ratios across the different patient phenotypes, a forest plot of the subgroup analyses is provided (Figure 6), with the comprehensive numerical outcomes summarized in Table 6. 

## 4. Discussion

### 4.1. Principal Findings and Methodological Strengths

This large-scale, propensity-matched analysis demonstrates that PELS is associated with a superior time-to-event profile regarding AUR compared to traditional open laminectomy. The most robust benefit was observed in our primary cohort of non-BPH males, where PELS was associated with a Hazard Ratio (HR) of 0.445 (95% CI: 0.284–0.697). A major methodological strength of this study is the deliberate definition of our postoperative observation window. Previous database studies utilizing catheterization billing codes to define AUR have frequently been limited by the inability to distinguish between an adverse retention event and routine, prophylactic intraoperative catheterization. By explicitly excluding the immediate 24 h postoperative period and analyzing events occurring up to 3 months postoperatively, our study effectively neutralizes this inherent coding bias. This rigorous temporal stratification ensures that the observed differences in AUR between the PELS and open laminectomy cohorts are reflective of true clinical complications rather than variations in standard perioperative workflow or anesthesiology practices. These findings are consistent with the hypotheses underlying the “tissue preservation” concept: open surgery requires extensive muscle stripping and prolonged retraction, which correlate with higher postoperative pain and increased opioid requirements [4,9]. By minimizing soft tissue trauma and potentially reducing the subsequent need for detrusor-suppressing narcotics, the PELS approach is associated with a lower continuous hazard of urinary retention [10]. Furthermore, the unique use of continuous saline irrigation in PELS is hypothesized to provide a distinct physiological advantage. While unmeasured in our dataset, it is hypothesized in the literature that this mechanism not only enhances visualization but may also assist in washing away local inflammatory mediators and reducing potential thermal injury to the nerve roots—factors that are thought to trigger sympathetically mediated retention [11]. This aligns with broader technical reviews of full-endoscopic spinal procedures (including transforaminal and interlaminar approaches), which highlight that abandoning extensive muscle splitting in favor of targeted percutaneous routes significantly alters local tissue stress and minimizes direct mechanical or thermal irritation to passing nerve roots [12].

### 4.2. Age-Related Nuances and the “Fragile Bladder”

Our age-stratified analysis reveals a complex interaction between surgical trauma and aging bladder physiology. The protective effect of PELS was most pronounced in the younger cohort (<70 years), which demonstrated a Hazard Ratio of 0.403 (95% CI: 0.217–0.746; Log-Rank *p* = 0.0028). In the older cohort (≥70 years), while the benefit remained statistically significant, the magnitude of protection attenuated slightly, with a Hazard Ratio of 0.574 (95% CI: 0.396–0.832; Log-Rank *p* = 0.0030). Furthermore, the elderly group failed the proportional hazards assumption (*p* = 0.0270), suggesting that the relative risk reduction provided by the endoscopic approach is not constant over the postoperative course. This likely reflects the “fragile bladder” phenomenon, where geriatric patients often have baseline detrusor underactivity [13,14]. In the aging lower urinary tract, a complex interplay exists between structural bladder outlet resistance and declining detrusor contractility [15]. This underlying autonomic and muscular vulnerability creates a clinical state where even subclinical perioperative stressors can precipitate sudden detrusor overdistention [16]. While PELS significantly reduces the initial surgical hazard, the accumulation of other time-dependent factors, such as immobility or delayed narcotic effects, may eventually erode this advantage in the frail population [17]. Recent analysis also confirms that age ≥ 65 is an independent predictor of retention even in minimally invasive procedures, supporting the notion that physiological frailty can override surgical technique [18].

### 4.3. BPH and Sex-Specific Trends

A critical nuance in our data is the attenuation of benefit in the BPH cohort. Although the PELS group showed a favorable trend with a Hazard Ratio of 0.505, the confidence interval crossed unity (95% CI: 0.235–1.086), and the difference failed to reach statistical significance (Log-Rank *p* = 0.0744).

Because this trend did not reach statistical significance, our study may be underpowered to draw definitive conclusions for this specific demographic. Alternatively, it may point to a competitive baseline phenomenon [19]: in patients with severe pre-existing outflow obstruction, such as clinical BPH, the underlying urologic pathology appears to drive the hazard rate to an extent that may obscure the technical benefits of a less invasive surgical approach. Similarly, in the female subgroup, the Hazard Ratio of 0.649 did not reach statistical significance (*p* = 0.1005). This outcome suggests that for female patients, the postoperative hazard of retention may be driven by different anatomical or clinical factors—such as pelvic floor dysfunction—that may be less sensitive to the choice of surgical technique compared to non-obstructed male cohorts [20].

### 4.4. Clinical Implications: A Risk-Stratified Approach

While retrospective data cannot establish definitive practice mandates, these observed associations may support the consideration of a risk-stratified approach within Enhanced Recovery After Surgery (ERAS) pathways [21]:

- Lower-Baseline Risk Cohorts (Younger Males): Utilizing PELS may represent a useful strategy to minimize surgical stress and narcotic use in this population, as this group consistently maintained the lowest hazard ratio within our data structure (HR = 0.403).
- Higher-Baseline Risk Cohorts (BPH/Elderly): Given that the relative surgical advantage appeared attenuated by physiological aging (HR = 0.574) or outpaced by baseline pathology, proactive clinical monitoring or the consideration of medical prophylaxis—such as perioperative alpha-blockers—warrants further investigation as a strategy to mitigate baseline hazards, irrespective of the surgical approach selected [22]. Furthermore, the implementation of standardized, evidence-based clinical practice guidelines for perioperative urologic care, including structured voiding trials and early mobilization, is critical to optimize recovery in these vulnerable cohorts [23].

## 5. Conclusions

This large-scale, propensity-matched analysis demonstrates that Percutaneous Endoscopic Lumbar Surgery (PELS) is associated with a significantly lower risk of postoperative acute urinary retention (AUR) compared to traditional open laminectomy. The protective benefit is most profound in males without benign prostatic hyperplasia (BPH) and patients under 70 years of age, in whom PELS reduced the instantaneous hazard of retention by approximately 55–60% (HR: 0.445 and 0.403, respectively). In these groups, the minimally invasive nature of PELS likely preserves autonomic function by minimizing tissue retraction and opioid requirements. However, this surgical advantage is not uniform across all demographics. In males with BPH, the benefit of PELS was attenuated and failed to reach statistical significance, suggesting that our study may be underpowered for this subgroup, or that baseline outflow obstruction heavily dictates retention risk regardless of surgical tissue preservation. Similarly, in the female cohort, although a favorable trend toward risk reduction was observed (HR: 0.649), this did not reach statistical significance (*p* = 0.1005). This suggests that retention in females may be driven by distinct anatomical factors—such as pelvic floor dysfunction—that are less sensitive to the reduction in surgical trauma provided by endoscopy. Furthermore, while elderly patients (>70 years) derived significant initial benefit, the protective effect diminished over time, likely due to age-related detrusor frailty. These observed associations warrant further investigation through prospective randomized trials. Standardized anesthesia, fluid management, and bladder scanning protocols are required to definitively establish clinical guidelines and determine if PELS should be prioritized for specific risk-stratified cohorts.

## 6. Limitations

The primary strength of this study lies in the utilization of the large-scale TriNetX federated network, which provided a robustly powered sample to analyze granular clinical subgroups that are frequently underpowered in single-center trials. However, several limitations inherent to retrospective administrative data registry analyses must be acknowledged. First, our reliance on ICD-10 diagnosis and procedural billing codes likely underestimates the true absolute incidence of postoperative retention. Mild, transient, or self-limiting episodes of voiding difficulty that were managed conservatively at the bedside—without the placement of a formal indwelling Foley catheter, straight catheterization, or an explicit electronic health record (EHR) diagnosis code for retention (ICD-10: R33)—may not be fully captured within this dataset. Second, due to the inherent structure of the TriNetX global network, several critical perioperative and intraoperative parameters were unavailable for extraction. These include:

- Unmeasured Perioperative Confounders: Longer surgical times are directly linked to prolonged exposure to anesthetic agents and increased fluid volumes. Granular data regarding operative duration, specific anesthesia subtypes (general vs. spinal), exact dosing of intraoperative long-acting opioids, IV fluid volumes, and mobilization times could not be mapped.
- Catheterization Protocols: Variations in institutional preferences regarding routine, proactive intraoperative indwelling catheter placement versus “void-on-demand” workflows could not be standardized.
- Disease Severity and Surgeon Factors: The exact number of surgical levels, radiographic stenosis severity, baseline neurological deficits, distinction between primary versus revision surgery, and individual surgeon volume were unavailable.
- Urologic Granularity: Our urologic stratification was based purely on diagnostic codes. Granular clinical metrics—including prostate volume, International Prostate Symptom Scores (IPSS), postvoid residuals, uroflowmetry, alpha-blocker use history, and urodynamic evidence of detrusor underactivity—were unavailable.
- Outcome Validation: While ICD-10 R33 and Foley insertion codes are standard proxies for retention, we do not possess a formally validated Positive Predictive Value (PPV) specifically for this TriNetX spine cohort.
- Baseline Imbalances and Hospital Clustering: Before matching, extreme baseline disparities were noted (e.g., race/ethnicity distributions). Because TriNetX aggregates data globally, these imbalances likely reflect geographic triage patterns or hospital-level coding biases. Furthermore, due to the privacy-preserving nature of the database, specific Healthcare Organization identifiers are blinded, preventing us from utilizing mixed-effects models to adjust for hospital-level clustering.

These unmeasured parameters represent undeniable sources of potential residual confounding. However, to strongly mitigate these administrative limitations, our statistical design employed a rigorous 1:1 propensity score matching model. By comprehensively balancing all available preoperative systemic comorbidities, metabolic disorders, cardiovascular status, and baseline urologic profiles across the cohorts, the comparative risk reduction and hazard trajectories observed in the PELS arm remain statistically robust and highly representative of real-world clinical practice. Furthermore, our deliberate choice to utilize a delayed observation window (from 24 h to 3 months postoperatively) explicitly insulated our primary outcome from the immediate confounding effects of routine intraoperative or recovery-room catheterization workflows. These findings represent real-world associations, not definitive causative mandates, and prospective randomized trials are required to establish clinical guidelines.

## Figures and Tables

**Table 1:** Patient characteristics of the Non-BPH Male cohort.

Characteristic	PELS (Before)	Open Surgery (Before)	*p*-Value (Before)	Std. Diff. (Before)	PELS (After)	Open Surgery (After)	*p*-Value (After)	Std. Diff. (After)
Age at Index	62.8 ± 12.9	59.3 ± 14.3	<0.001	0.263	61.0 ± 13.2	61.0 ± 14.3	0.997	<0.001
White	427 (12.6%)	42,393 (80.4%)	<0.001	1.850	427 (17.8%)	427 (17.8%)	1.000	<0.001
Black or African American	38 (1.1%)	4195 (8.0%)	<0.001	0.333	38 (1.6%)	37 (1.5%)	0.907	0.003
Asian	741 (21.9%)	1360 (2.6%)	<0.001	0.618	673 (28.1%)	644 (26.9%)	0.348	0.027
Not Hispanic or Latino	1090 (32.3%)	41,970 (79.6%)	<0.001	1.083	1023 (42.7%)	982 (41.0%)	0.230	0.035
Unknown Ethnicity	2267 (67.1%)	7580 (14.4%)	<0.001	1.273	1351 (56.4%)	1393 (58.2%)	0.220	0.035
Cerebrovascular diseases	143 (4.2%)	2549 (4.8%)	0.115	0.029	100 (4.2%)	73 (3.0%)	0.037	0.060
Chronic rheumatic heart diseases	14 (0.4%)	725 (1.4%)	<0.001	0.102	11 (0.5%)	10 (0.4%)	0.827	0.006
Diabetes mellitus	585 (17.3%)	9083 (17.2%)	0.879	0.003	406 (17.0%)	353 (14.7%)	0.036	0.061
Diseases of the nervous system	686 (20.3%)	26,982 (51.2%)	<0.001	0.680	639 (26.7%)	615 (25.7%)	0.430	0.023
Diseases of the respiratory system	458 (13.6%)	12,628 (23.9%)	<0.001	0.268	354 (14.8%)	327 (13.7%)	0.264	0.032
Hypertensive diseases	1103 (32.7%)	22,244 (42.2%)	<0.001	0.198	782 (32.7%)	736 (30.7%)	0.153	0.041
Ischemic heart diseases	284 (8.4%)	7095 (13.5%)	<0.001	0.162	225 (9.4%)	217 (9.1%)	0.690	0.012
Metabolic disorders	521 (15.4%)	18,559 (35.2%)	<0.001	0.467	470 (19.6%)	489 (20.4%)	0.493	0.020
Overweight, obesity and other hyperalimentation	71 (2.1%)	9188 (17.4%)	<0.001	0.534	71 (3.0%)	73 (3.0%)	0.866	0.005
Tobacco use	11 (0.3%)	2230 (4.2%)	<0.001	0.264	11 (0.5%)	12 (0.5%)	0.834	0.006

**Table 2:** Patient characteristics of Male patients with BPH.

Characteristic	PELS (Before)	Open Surgery (Before)	*p*-Value (Before)	Std. Diff. (Before)	PELS (After)	Open Surgery (After)	*p*-Value (After)	Std. Diff. (After)
Age at Index	71.1 ± 8.4	69.9 ± 9.0	0.006	0.140	71.6 ± 8.1	71.6 ± 9.8	0.971	0.003
White	32 (7.4%)	5871 (81.3%)	<0.001	2.224	32 (13.8%)	35 (15.1%)	0.692	0.037
Black or African American	10 (2.3%)	662 (9.2%)	<0.001	0.297	10 (4.3%)	10 (4.3%)	1.000	<0.001
Asian	151 (35.1%)	254 (3.5%)	<0.001	0.873	97 (41.8%)	101 (43.5%)	0.707	0.035
Not Hispanic or Latino	184 (42.8%)	5961 (82.6%)	<0.001	0.902	130 (56.0%)	132 (56.9%)	0.851	0.017
Unknown Ethnicity	243 (56.5%)	909 (12.6%)	<0.001	1.041	99 (42.7%)	100 (43.1%)	0.925	0.009
Cerebrovascular diseases	60 (14.0%)	1108 (15.3%)	0.435	0.039	36 (15.5%)	27 (11.6%)	0.223	0.113
Chronic rheumatic heart diseases	10 (2.3%)	373 (5.2%)	0.009	0.150	10 (4.3%)	10 (4.3%)	1.000	<0.001
Diabetes mellitus	155 (36.0%)	2555 (35.4%)	0.783	0.014	90 (38.8%)	82 (35.3%)	0.442	0.071
Diseases of the nervous system	198 (46.0%)	5671 (78.6%)	<0.001	0.712	140 (60.3%)	134 (57.8%)	0.571	0.053
Diseases of the respiratory system	162 (37.7%)	3624 (50.2%)	<0.001	0.254	100 (43.1%)	100 (43.1%)	1.000	<0.001
Hypertensive diseases	288 (67.0%)	5767 (79.9%)	<0.001	0.295	169 (72.8%)	163 (70.3%)	0.537	0.057
Ischemic heart diseases	81 (18.8%)	2670 (37.0%)	<0.001	0.413	65 (28.0%)	61 (26.3%)	0.676	0.039
Metabolic disorders	149 (34.7%)	5642 (78.2%)	<0.001	0.976	137 (59.1%)	130 (56.0%)	0.511	0.061
Overweight, obesity and other hyperalimentation	12 (2.8%)	2357 (32.6%)	<0.001	0.850	12 (5.2%)	12 (5.2%)	1.000	<0.001
Tobacco use	10 (2.3%)	433 (6.0%)	0.002	0.185	10 (4.3%)	10 (4.3%)	1.000	<0.001

**Table 3:** Patient characteristics of the Age < 70 cohort.

Characteristic	PELS (Before)	Open Surgery (Before)	*p*-Value (Before)	Std. Diff. (Before)	PELS (After)	Open Surgery (After)	*p*-Value (After)	Std. Diff. (After)
Age at Index	54.4 ± 9.7	51.4 ± 11.5	<0.001	0.286	52.9 ± 10.4	52.2 ± 11.1	0.046	0.059
Male	1836 (55.5%)	34,164 (57.6%)	0.017	0.042	1325 (58.0%)	1358 (59.4%)	0.322	0.029
White	302 (9.1%)	46,101 (77.7%)	<0.001	1.917	302 (13.2%)	312 (13.6%)	0.664	0.013
Black or African American	38 (1.1%)	6232 (10.5%)	<0.001	0.408	38 (1.7%)	37 (1.6%)	0.907	0.003
Asian	864 (26.1%)	1290 (2.2%)	<0.001	0.732	659 (28.8%)	650 (28.4%)	0.768	0.009
Not Hispanic or Latino	1096 (33.1%)	46,136 (77.8%)	<0.001	1.006	894 (39.1%)	848 (37.1%)	0.161	0.041
Unknown Ethnicity	2189 (66.2%)	9026 (15.2%)	<0.001	1.213	1369 (59.9%)	1413 (61.8%)	0.182	0.039
Benign prostatic hyperplasia	113 (3.4%)	2065 (3.5%)	0.841	0.004	68 (3.0%)	61 (2.7%)	0.532	0.018
Cerebrovascular diseases	103 (3.1%)	2187 (3.7%)	0.087	0.032	68 (3.0%)	55 (2.4%)	0.235	0.035
Chronic rheumatic heart diseases	10 (0.3%)	689 (1.2%)	<0.001	0.101	10 (0.4%)	10 (0.4%)	1.000	<0.001
Diabetes mellitus	536 (16.2%)	9360 (15.8%)	0.518	0.011	352 (15.4%)	276 (12.1%)	0.001	0.097
Diseases of the nervous system	814 (24.6%)	34,026 (57.4%)	<0.001	0.707	732 (32.0%)	680 (29.7%)	0.096	0.049
Diseases of the respiratory system	542 (16.4%)	18,847 (31.8%)	<0.001	0.366	408 (17.8%)	367 (16.1%)	0.106	0.048
Hypertensive diseases	974 (29.4%)	22,833 (38.5%)	<0.001	0.192	634 (27.7%)	578 (25.3%)	0.061	0.056
Ischemic heart diseases	194 (5.9%)	4969 (8.4%)	<0.001	0.098	144 (6.3%)	128 (5.6%)	0.317	0.030
Metabolic disorders	447 (13.5%)	20,227 (34.1%)	<0.001	0.498	404 (17.7%)	383 (16.8%)	0.411	0.024
Overweight, obesity and other hyperalimentation	102 (3.1%)	14,217 (24.0%)	<0.001	0.641	102 (4.5%)	99 (4.3%)	0.829	0.006
Tobacco use	14 (0.4%)	3633 (6.1%)	<0.001	0.325	14 (0.6%)	16 (0.7%)	0.714	0.011

**Table 4:** Patient characteristics of the Age ≥ 70 cohort.

Characteristic	PELS (Before)	Open Surgery (Before)	*p*-Value (Before)	Std. Diff. (Before)	PELS (After)	Open Surgery (After)	*p*-Value (After)	Std. Diff. (After)
Age at Index	73.5 ± 6.4	73.1 ± 5.9	<0.001	0.061	73.3 ± 6.5	73.2 ± 6.1	0.635	0.014
Male	1943 (43.2%)	24,613 (53.7%)	<0.001	0.211	1146 (49.0%)	1192 (51.0%)	0.178	0.039
White	514 (11.4%)	38,281 (83.6%)	<0.001	2.088	514 (22.0%)	527 (22.6%)	0.648	0.013
Black or African American	36 (0.8%)	2981 (6.5%)	<0.001	0.308	36 (1.5%)	29 (1.2%)	0.382	0.026
Asian	967 (21.5%)	1493 (3.3%)	<0.001	0.577	792 (33.9%)	751 (32.1%)	0.202	0.037
Not Hispanic or Latino	1413 (31.4%)	37,910 (82.7%)	<0.001	1.212	1239 (53.0%)	1187 (50.8%)	0.128	0.045
Unknown Ethnicity	3065 (68.2%)	6077 (13.3%)	<0.001	1.349	1083 (46.3%)	1137 (48.7%)	0.114	0.046
Benign prostatic hyperplasia	347 (7.7%)	4367 (9.5%)	<0.001	0.064	179 (7.7%)	184 (7.9%)	0.785	0.008
Cerebrovascular diseases	319 (7.1%)	4070 (8.9%)	<0.001	0.066	164 (7.0%)	138 (5.9%)	0.122	0.045
Chronic rheumatic heart diseases	22 (0.5%)	1344 (2.9%)	<0.001	0.189	21 (0.9%)	11 (0.5%)	0.076	0.052
Diabetes mellitus	980 (21.8%)	10,293 (22.5%)	0.314	0.016	492 (21.1%)	473 (20.2%)	0.492	0.020
Diseases of the nervous system	1156 (25.7%)	24,495 (53.5%)	<0.001	0.591	791 (33.8%)	732 (31.3%)	0.066	0.054
Diseases of the respiratory system	777 (17.3%)	12,980 (28.3%)	<0.001	0.265	478 (20.5%)	453 (19.4%)	0.360	0.027
Hypertensive diseases	1998 (44.5%)	24,972 (54.5%)	<0.001	0.202	1034 (44.2%)	1002 (42.9%)	0.345	0.028
Ischemic heart diseases	433 (9.6%)	8974 (19.6%)	<0.001	0.285	289 (12.4%)	267 (11.4%)	0.320	0.029
Metabolic disorders	925 (20.6%)	21,533 (47.0%)	<0.001	0.582	698 (29.9%)	716 (30.6%)	0.567	0.017
Overweight, obesity and other hyperalimentation	75 (1.7%)	7886 (17.2%)	<0.001	0.551	74 (3.2%)	97 (4.2%)	0.073	0.052
Tobacco use	10 (0.2%)	910 (2.0%)	<0.001	0.169	10 (0.4%)	11 (0.5%)	0.827	0.006

**Table 5:** Patient characteristics of the Female cohort.

Characteristic	PELS (Before)	Open Surgery (Before)	*p*-Value (Before)	Std. Diff. (Before)	PELS (After)	Open Surgery (After)	*p*-Value (After)	Std. Diff. (After)
Age at Index	67.0 ± 11.7	61.5 ± 14.5	<0.001	0.417	64.7 ± 12.7	64.2 ± 14.5	0.184	0.041
White	360 (9.0%)	37,062 (80.0%)	<0.001	2.045	360 (16.8%)	368 (17.2%)	0.745	0.010
Black or African American	33 (0.8%)	4465 (9.6%)	<0.001	0.404	33 (1.5%)	32 (1.5%)	0.901	0.004
Asian	947 (23.5%)	1215 (2.6%)	<0.001	0.653	742 (34.6%)	743 (34.7%)	0.974	<0.001
Not Hispanic or Latino	1246 (31.0%)	37,098 (80.1%)	<0.001	1.137	1042 (48.6%)	1016 (47.4%)	0.427	0.024
Unknown Ethnicity	2760 (68.6%)	6731 (14.5%)	<0.001	1.313	1085 (50.6%)	1113 (51.9%)	0.392	0.026
Cerebrovascular diseases	225 (5.6%)	2827 (6.1%)	0.195	0.022	120 (5.6%)	88 (4.1%)	0.023	0.070
Chronic rheumatic heart diseases	15 (0.4%)	1024 (2.2%)	<0.001	0.163	14 (0.7%)	10 (0.5%)	0.413	0.025
Diabetes mellitus	787 (19.6%)	8510 (18.4%)	0.061	0.031	365 (17.0%)	343 (16.0%)	0.366	0.028
Diseases of the nervous system	1103 (27.4%)	26,901 (58.1%)	<0.001	0.652	786 (36.7%)	737 (34.4%)	0.118	0.048
Diseases of the respiratory system	711 (17.7%)	16,255 (35.1%)	<0.001	0.403	461 (21.5%)	418 (19.5%)	0.104	0.050
Hypertensive diseases	1601 (39.8%)	20,800 (44.9%)	<0.001	0.103	777 (36.3%)	761 (35.5%)	0.610	0.016
Ischemic heart diseases	269 (6.7%)	4686 (10.1%)	<0.001	0.124	163 (7.6%)	142 (6.6%)	0.212	0.038
Metabolic disorders	715 (17.8%)	18,531 (40.0%)	<0.001	0.506	540 (25.2%)	548 (25.6%)	0.779	0.009
Overweight, obesity and other hyperalimentation	96 (2.4%)	11,008 (23.8%)	<0.001	0.669	93 (4.3%)	94 (4.4%)	0.940	0.002
Tobacco use	11 (0.3%)	1963 (4.2%)	<0.001	0.269	11 (0.5%)	19 (0.9%)	0.143	0.045

**Table 6:** Post-operative acute urinary retention by subgroup: PELS versus traditional open surgery. AUR-free survival at 90 days (with 95% CI), event counts, hazard ratios, and log-rank *p*-values for each pre-specified subgroup. Highlighted rows denote subgroups with a statistically significant between-group difference.

Figure	Subgroup	PELS Events/N	PELS AUR-Free Survival at 90 d, % (95% CI)	Open Surgery Events/N	Open Surgery AUR-Free Survival at 90 d, % (95% CI)	HR (95% CI)	Log-Rank *p*
1	Non-BPH male	27/2394	98.7 (98.1–99.1)	65/2394	97.1 (96.3–97.7)	0.44 (0.28–0.70)	<0.001
2	BPH male	10/232	96.4 (93.0–98.2)	19/232	91.5 (86.9–94.5)	0.51 (0.23–1.09)	0.07
3	Age < 70 years	14/2286	99.3 (98.8–99.6)	36/2286	98.3 (97.7–98.8)	0.40 (0.22–0.75)	<0.001
4	Age ≥ 70 years	43/2337	97.9 (97.2–98.4)	80/2337	96.4 (95.5–97.1)	0.57 (0.40–0.83)	<0.001
5	Female	23/2143	98.8 (98.2–99.2)	37/2143	98.2 (97.5–98.7)	0.65 (0.39–1.09)	0.10

AUR, acute urinary retention; PELS, percutaneous endoscopic lumbar surgery; HR, hazard ratio; CI, confidence interval. HR < 1 favours PELS. Survival percentages and confidence intervals are reported to one decimal place, and hazard ratios to two decimal places; *p*-values below 0.001 are reported as < 0.001. Corresponding Kaplan–Meier curves are shown in Figure 1, Figure 2, Figure 3, Figure 4 and Figure 5.

## Data Availability

The data used in this study were collected from the TriNetX Global Health Research Network. Data may be made available upon reasonable request to the corresponding author, subject to TriNetX network data sharing agreements.

## Supplementary Materials

The following supporting information can be downloaded at: https://www.mdpi.com/article/10.3390/jcm15124519/s1, Supplementary Methods: Methodological details on restricted mean survival time (RMST), variance estimation, and multiplicity control; Table S1: Restricted mean survival time (RMST) difference at τ = 90 days, by subgroup; Table S2: Log-rank *p*-values for the five pre-specified subgroups before and after Bonferroni correction (α = 0.01); Supplementary Interpretation: Interpretation of RMST and multiplicity control results; STROBE Checklist: STROBE Statement checklist of items included in reports of observational studies.

## Abbreviations

The following abbreviations are used in this manuscript:

AUR	Acute urinary retention
BPH	Benign prostatic hyperplasia
CI	Confidence intervals
ERAS	Enhanced Recovery After Surgery
HR	Hazard Ratio
IPSS	International Prostate Symptom Scores
MIS	Minimally invasive surgery
ODI	Oswestry Disability Index
OR	Odds Ratio
PELS	Percutaneous endoscopic lumbar surgery
PSM	Propensity score matching
RR	Risk Ratio
SMD	Standardized mean differences
VAS	Visual Analog Scale
