# Supplementary material for: Impact of Percutaneous Endoscopic Decompression Versus Open Laminectomy on Postoperative Acute Urinary Retention: A Large-Scale Real-World Data Analysis
Source: J Clin Med. 2026 Jun 11;15(12):4519. doi: 10.3390/jcm15124519 (PMC13301296; doi:10.3390/jcm15124519)
Supplement: Supplementary file 1 [file jcm-15-04519-s001.zip › STROBE-checklist.pdf]

STROBE Statement—checklist of items that should be included in reports of observational studies

| | Item No. | Recommendation | Section. | Relevant text from manuscript |
|---|---|---|---|---|
| **Title and abstract** | 1 | (*a*) Indicate the study's design with a commonly used term in the title or the abstract | Abstract | A retrospective, propensity score-matched analysis was conducted |
| | | (*b*) Provide in the abstract an informative and balanced summary of what was done and what was found | Abstract | The abstract encompasses structured Background/Objectives, Methods, Results, and Conclusions sections summarizing the study |
| **Introduction** | | | | |
| Background/rationale | 2 | Explain the scientific background and rationale for the investigation being reported | 1. Introduction | Acute urinary retention (AUR) is a frequent early postoperative complication following lumbar spine surgery... Surgical invasiveness is hypothesized to play a pivotal role in this mechanism. |
| Objectives | 3 | State specific objectives, including any prespecified hypotheses | 1. Introduction | The objective of this study is to compare the incidence of postoperative acute urinary retention between traditional open lumbar surgery and percutaneous endoscopic lumbar surgery. |
| **Methods** | | | | |
| Study design | 4 | Present key elements of study design early in the paper | 2.1 Data Source and Study | A retrospective, propensity score-matched analysis was |

| | | | | |
|---|---|---|---|---|
| | | | Design | conducted using the TriNetX Global Health Research Network... |
| Setting | 5 | Describe the setting, locations, and relevant dates, including periods of recruitment, exposure, follow-up, and data collection | 2.1 & 2.2 | The data used in this study was collected on January 7, 2026, from the TriNetX Global Health Research Network... Adult patients... between January 1, 2015, and December 31, 2024... |
| Participants | 6 | (*a*) *Cohort study*—Give the eligibility criteria, and the sources and methods of selection of participants. Describe methods of follow-up<br>*Case-control study*—Give the eligibility criteria, and the sources and methods of case ascertainment and control selection. Give the rationale for the choice of cases and controls<br>*Cross-sectional study*—Give the eligibility criteria, and the sources and methods of selection of participants | 2.2 Study Population | Adult patients (aged >18 years) who underwent lumbar spine decompression procedures... systematically excluded any patient with a documented historical diagnosis of neurogenic bladder, baseline preoperative urinary retention, or chronic indwelling catheterization... |
| | | (*b*) *Cohort study*—For matched studies, give matching criteria and number of exposed and unexposed<br>*Case-control study*—For matched studies, give matching criteria and the number of controls per case | 2.2 & 2.5 | Within each subpopulation, 1:1 nearest-neighbor propensity score matching (PSM) was executed." "Match performance was evaluated using a 1:1 nearest-neighbor matching algorithm with a strict caliper width of 0.1 pooled standard deviations. |
| Variables | 7 | Clearly define all outcomes, exposures, predictors, potential confounders, and effect modifiers. Give diagnostic criteria, if applicable | 2.3 & 2.4 | The primary outcome was the incidence of acute urinary retention (AUR)." "Patients |

| | | | | |
|---|---|---|---|---|
| | | | | were categorized into two mutually exclusive surgical exposure cohorts based on a validated, granular billing and procedural coding strategy. |
| Data sources/ measurement | 8* | For each variable of interest, give sources of data and details of methods of assessment (measurement). Describe comparability of assessment methods if there is more than one group | 2.3 & 2.4 | AUR was identified by the presence of the diagnostic code for retention (ICD-10: R33) and/or procedural codes indicating Foley catheter insertion (CPT 51702, 51703)." (Surgical cohorts defined by specific CPT and ICD-10-PCS codes. ) |
| Bias | 9 | Describe any efforts to address potential sources of bias | 2.4 & 2.5 | Any retention codes or catheterizations documented on the day of surgery (Day 0 to <24 hours) were excluded... ensuring only true, symptomatic postoperative retention events were captured." "Propensity score matching (PSM) was implemented to balance baseline covariates... |
| Study size | 10 | Explain how the study size was arrived at | 2.2 Study Population | Because this study utilized a pre-existing federated database, the sample size was determined by the total available patient cohort that met our strict eligibility criteria... rather than an a priori power calculation. |

Continued on next page

| | | | | |
|---|---|---|---|---|
| Quantitative variables | 11 | Explain how quantitative variables were handled in the analyses. If applicable, describe which groupings were chosen and why | 2.3 & 2.5 | Patients were stratified into those aged <70 years and those aged ≥70 years." "For normally distributed continuous data, independent two-sample t-tests were performed, while the Mann–Whitney U test was designated for non-normally distributed metrics. |
| Statistical methods | 12 | (*a*) Describe all statistical methods, including those used to control for confounding | 2.5 Statistical Analysis | To control for confounding and selection bias inherent to retrospective observational studies, propensity score matching (PSM) was implemented to balance baseline covariates... |
| | | (*b*) Describe any methods used to examine subgroups and interactions | 2.3 Exposure Definitions | To evaluate the differential impact of surgical technique across vulnerable populations, the cohort was stratified into three distinct categories: Urologic Status... Age... Sex... |
| | | (*c*) Explain how missing data were addressed | 2.2 Study Population | Patients who lacked at least 30 days of continuous post-operative health record data within the federated network were excluded to minimize attrition bias... |
| | | (*d*) *Cohort study*—If applicable, explain how loss to follow-up was addressed<br>*Case-control study*—If applicable, explain how matching of cases and controls was addressed<br>*Cross-sectional study*—If applicable, describe analytical methods taking account of sampling strategy | 2.5 Statistical Analysis | Loss to follow-up was mitigated by requiring a minimum of 30 days continuous data. "Match performance was evaluated using a 1:1 nearest-neighbor matching algorithm with a strict caliper width |

| | | | | |
|---|---|---|---|---|
| | | | | of 0.1 pooled standard deviations. |
| | | (*e*) Describe any sensitivity analyses | 2.5 Statistical Analysis | Finally, to quantify the potential impact of unmeasured confounding, we calculated the E-value for our primary cohort. |
| **Results** | | | | |
| Participants | 13* | (a) Report numbers of individuals at each stage of study—eg numbers potentially eligible, examined for eligibility, confirmed eligible, included in the study, completing follow-up, and analysed | 3.1 & Tables 1-5 | Non-BPH Male Cohort: The matched analysis included approximately 2,398 patients per group... BPH Male Cohort: This high-risk subgroup (approx. N=232 per group)... |
| | | (b) Give reasons for non-participation at each stage | 2.2 Study Population | The selection process followed a structured, sequential attrition pipeline to isolate patients with homogenous baseline characteristics… |
| | | (c) Consider use of a flow diagram | N/A | A formal flow diagram is absent, but the sequential filtering process is outlined in the text. |
| Descriptive data | 14* | (a) Give characteristics of study participants (eg demographic, clinical, social) and information on exposures and potential confounders | 3.1 & Tables 1-5 | Baseline characteristics for these groups prior to and following propensity score matching (PSM) are detailed in Tables 1–5. |
| | | (b) Indicate number of participants with missing data for each variable of interest | 2.2 Study Population | Data attrition due to missingness was handled upfront by excluding patients who lacked continuous post-operative health record data. |
| | | (c) *Cohort study*—Summarise follow-up time (eg, average and total amount) | 2.4 Primary Outcome | The observation window was strictly set from 24 hours to 3 months following the index |

| | | | | |
|---|---|---|---|---|
| | | | | surgery. |
| Outcome data | 15* | *Cohort study*—Report numbers of outcome events or summary measures over time | 3.2 & Table 6 | Total event counts, AUR-free survival percentages, hazard ratios, and log-rank p-values for all pre-specified subgroups are systematically reported in Table 6. |
| | | *Case-control study*—Report numbers in each exposure category, or summary measures of exposure | | |
| | | *Cross-sectional study*—Report numbers of outcome events or summary measures | | |
| Main results | 16 | (*a*) Give unadjusted estimates and, if applicable, confounder-adjusted estimates and their precision (eg, 95% confidence interval). Make clear which confounders were adjusted for and why they were included | 3.2.1 | Patients in the PELS group experienced... a 55.5% lower instantaneous hazard (HR: 0.445; 95% CI: 0.284–0.697) compared to the Traditional open surgery. |
| | | (*b*) Report category boundaries when continuous variables were categorized | 2.3 Exposure Definitions | Patients were stratified into those aged <70 years and those aged ≥70 years. |
| | | (*c*) If relevant, consider translating estimates of relative risk into absolute risk for a meaningful time period | 3.2.1 | PELS cohort demonstrated a significantly lower incidence of AUR... with absolute risks of 1.128% and 2.715%, respectively (Risk Difference: -1.587%; p < 0.0001). |

Continued on next page

| | | | | |
|---|---|---|---|---|
| Other analyses | 17 | Report other analyses done—eg analyses of subgroups and interactions, and sensitivity analyses | 3.2 | Reports subgroup outcomes for Non-BPH Males , BPH Patients , Age < 70 , Age ≥70 , and Females , alongside sensitivity analysis via E-value. |
| **Discussion** | | | | |
| Key results | 18 | Summarise key results with reference to study objectives | 4.1 Principal Findings | This large-scale, propensity-matched analysis demonstrates that PELS is associated with a superior time-to-event profile regarding AUR compared to traditional open laminectomy. |
| Limitations | 19 | Discuss limitations of the study, taking into account sources of potential bias or imprecision. Discuss both direction and magnitude of any potential bias | 6. Limitations | Explicitly discusses limitations regarding reliance on ICD-10/CPT codes , unmeasured perioperative confounders (e.g., fluids, anesthesia duration) , lack of urologic granularity , and hospital clustering. |
| Interpretation | 20 | Give a cautious overall interpretation of results considering objectives, limitations, multiplicity of analyses, results from similar studies, and other relevant evidence | 5. Conclusions | These observed associations warrant further investigation through prospective, randomized trials. Standardized anesthesia, fluid management, and bladder scanning protocols are required to definitively establish clinical guidelines... |
| Generalisability | 21 | Discuss the generalisability (external validity) of the study results | 6. Limitations | Notes the advantage of the large-scale TriNetX network providing a "robustly powered sample" and outcomes that remain "highly representative of real-world clinical |

| | | | | |
|---|---|---|---|---|
| | | | | practice. |
| **Other information** | | | | |
| Funding | 22 | Give the source of funding and the role of the funders for the present study and, if applicable, for the original study on which the present article is based | 7. Patents | Funding: This research received no external funding |

*Give information separately for cases and controls in case-control studies and, if applicable, for exposed and unexposed groups in cohort and cross-sectional studies.

**Note:** An Explanation and Elaboration article discusses each checklist item and gives methodological background and published examples of transparent reporting. The STROBE checklist is best used in conjunction with this article (freely available on the Web sites of PLoS Medicine at http://www.plosmedicine.org/, Annals of Internal Medicine at http://www.annals.org/, and Epidemiology at http://www.epidem.com/). Information on the STROBE Initiative is available at www.strobe-statement.org.
