# Supplementary material for: Impact of Percutaneous Endoscopic Decompression Versus Open Laminectomy on Postoperative Acute Urinary Retention: A Large-Scale Real-World Data Analysis
Source: J Clin Med. 2026 Jun 11;15(12):4519. doi: 10.3390/jcm15124519 (PMC13301296; doi:10.3390/jcm15124519)
Supplement: Supplementary file 1 [file jcm-15-04519-s001.zip › Supplementary Material.pdf]

Supplementary Material

Comparison of percutaneous endoscopic lumbar surgery (PELS) versus *traditional open surgery: supplementary statistical analyses addressing the proportional-hazards assumption and multiplicity in subgroup testing.*

1. Supplementary Methods

1.1. Restricted mean survival time (RMST). The proportional-hazards (PH) assumption was assessed for every subgroup using the Schoenfeld residual test. In subgroups where the assumption was violated ($p < 0.05$), a single global hazard ratio (HR) may misrepresent the time-varying treatment effect; we therefore additionally report the restricted mean survival time (RMST), defined as the area under the Kaplan–Meier (KM) curve up to a fixed horizon τ. This is a model-free measure that does not require the PH assumption and is interpreted directly as the mean event-free time accrued over the follow-up window. A horizon of $\tau = 90$ days was pre-specified to match the post-operative observation period for acute urinary retention (AUR).

1.2. Variance estimation. Patient-level number-at-risk tables were not exportable from the source platform; however, the KM graph export provides the survival probability and its 95% confidence interval (CI) at each day. Because these intervals are computed using Greenwood's formula, the pointwise standard error was recovered as $SE(t) = (CI\_upper - CI\_lower) / (2 \times 1.96)$. These pointwise standard errors were propagated through the RMST integral to obtain the standard error of each cohort's RMST and, by combination, the RMST difference, its 95% CI, and a two-sided p-value. The propagation used a conservative positive-autocorrelation approximation, which errs toward wider intervals. This approach is consistent with the area-under-the-KM-curve definition of RMST described by Uno et al. (J Clin Oncol 2014).

1.3. Multiplicity control. Five pre-specified subgroups were evaluated. To control the family-wise error rate, the Bonferroni correction was applied to the log-rank p-values, giving an adjusted significance threshold of $\alpha = 0.05 / 5 = 0.01$. Subgroups were defined a priori and were not selected post hoc.

2. Supplementary Results

Table S1. Restricted mean survival time (RMST) difference at $\tau = 90$ days by subgroup. Positive values favour PELS (more AUR-free days). Highlighted rows indicate subgroups in which the proportional-hazards assumption was violated and for which RMST is the preferred effect measure.

Subgroup	PH test p	PH violated	RMST diff. (days)	95% CI (days)	p-value
**Non-BPH male**	0.0317	Yes	+1.31	+0.60 to +2.02	0.0003
**Age > 70 y**	0.0270	Yes	+1.30	+0.48 to +2.11	0.0019
BPH male	0.2493	No	+3.97	+0.15 to +7.79	0.0417
Age ≤ 70 y	0.7299	No	+0.75	+0.21 to +1.30	0.0069
Female	0.0630	No	+0.55	−0.08 to +1.18	0.0888

RMST, restricted mean survival time; PH, proportional hazards; CI, confidence interval; BPH, benign prostatic hyperplasia. PH violation is defined as Schoenfeld residual test $p < 0.05$.

Table S2. Log-rank p-values for the five pre-specified subgroups before and after Bonferroni correction (α = 0.01). Highlighted rows remain statistically significant after correction.

Subgroup	HR	Log-rank p (raw)	Bonferroni-adjusted p	Significant (α = 0.01)
**Non-BPH male**	0.44	0.000279	0.0014	Yes
**Age > 70 y**	0.57	0.002976	0.0149	Yes
**Age ≤ 70 y**	0.40	0.002793	0.0140	Yes
BPH male	0.51	0.074433	0.3722	No
Female	0.65	0.100538	0.5027	No

HR, hazard ratio. Bonferroni-adjusted p = raw p × 5 (capped at 1.0). Significance threshold α = 0.05 / 5 = 0.01.

3. Interpretation

In the two subgroups with a violated PH assumption (non-BPH male and age > 70 years), the RMST analysis confirmed a benefit of PELS that does not depend on a constant hazard ratio: PELS patients remained AUR-free for approximately 1.3 additional days on average over the 90-day window (non-BPH male, +1.31 days, 95% CI +0.60 to +2.02, p = 0.0003; age > 70 years, +1.30 days, 95% CI +0.48 to +2.11, p = 0.0019). After Bonferroni correction, three of the five subgroups (non-BPH male, age ≤ 70 years, and age > 70 years) retained statistical significance, while the BPH male and female subgroups did not. These analyses confirm the robustness of the primary findings to both the PH assumption concern and the multiplicity of subgroup testing.
